# HIV-1 exposure triggers autophagic degradation of stathmin and hyperstabilization of microtubules to disrupt epithelial cell junctions

**DOI:** 10.1038/s41392-020-0175-1

**Published:** 2020-06-19

**Authors:** Wei Xie, Dengwen Li, Dan Dong, Yuanyuan Li, You Zhang, Liangwei Duan, Xinqi Liu, Wenxiang Meng, Min Liu, Jun Zhou

**Affiliations:** 1 Institute of Biomedical Sciences, Shandong Provincial Key Laboratory of Animal Resistance Biology, Collaborative Innovation Center of Cell Biology in Universities of Shandong, College of Life Sciences, Shandong Normal University, Jinan, Shandong 250014 China; 2 State Key Laboratory of Medicinal Chemical Biology, College of Life Sciences, Nankai University, Tianjin, 300071 China; 3 State Key Laboratory of Molecular Developmental Biology, Institute of Genetics and Developmental Biology, Chinese Academy of Sciences, Beijing, 100101 China

**Keywords:** Infection, Cell biology

**Dear Editor,**

Human immunodeficiency virus type 1 (HIV-1) infection is mainly initiated on mucosal epithelial surfaces. Exposure to HIV-1 impairs the mucosal epithelial barrier, allowing viral transmission across the mucosal epithelium.^1^ Interaction of HIV-1 envelope glycoprotein gp120 with its primary receptor, cluster of differentiation 4 (CD4), and chemokine coreceptors has been implicated in the disruption of epithelial cell junctions upon HIV-1 exposure.^1^ However, the molecular details and underlying mechanisms of this disruption remain elusive.

To investigate the mechanisms, we purified recombinant gp120, which was functional, as indicated by its efficient interaction with CD4 (Supplementary Data Fig. S1a–c). As T84 human colon cancer cells gradually achieved confluency, the transepithelial electrical resistance (TEER) was increased but was significantly decreased by the addition of gp120 to the culture (Supplementary Data Fig. S1d). The increase in epithelial permeability by gp120 was confirmed by the measurement of paracellular permeability (Supplementary Data Fig. S1e). By examining the localization of the tight junction marker zona occludens 1 (ZO-1) and the adherens junction marker E-cadherin, we found that gp120 impaired the integrity of tight and adherens junctions in T84, SW480, and Caco-2 human colon cancer cells (Fig. 1a, b) and HOEC human oral epithelial cells (Supplementary Data Fig. S1f, g).

**Fig. 1:**
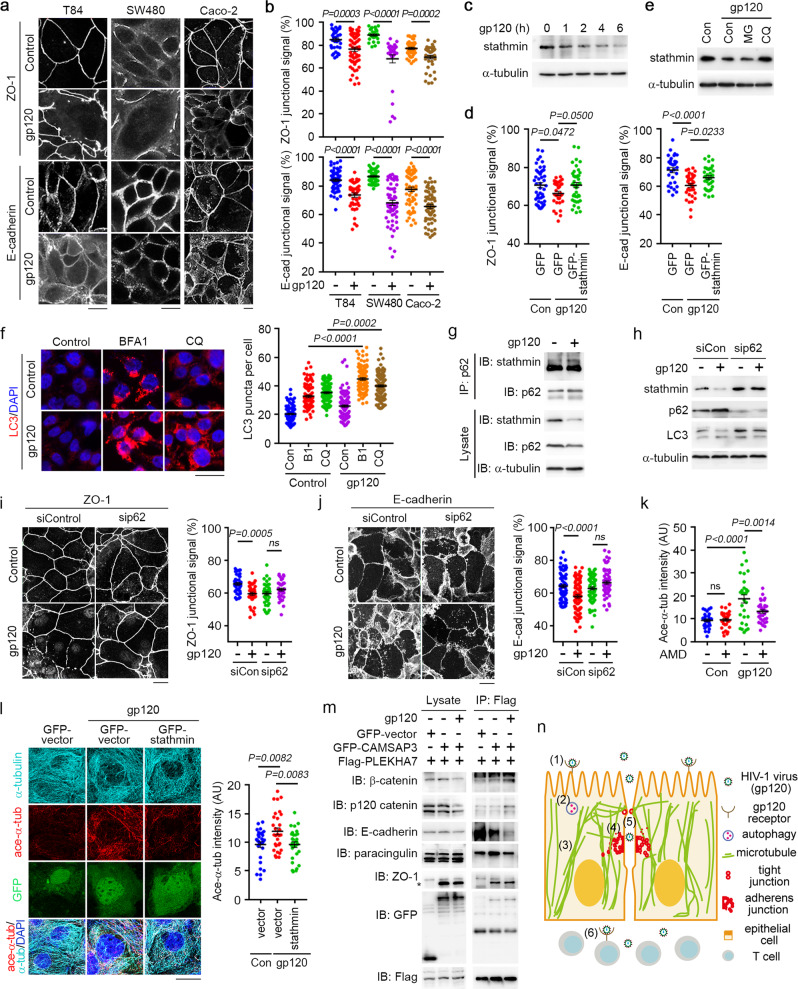
Autophagic degradation of stathmin and hyperstabilization of microtubules mediate the perturbation of mucosal epithelial cell junctions upon HIV-1 exposure. **a** Immunofluorescence staining of ZO-1 and E-cadherin in T84, SW480, and Caco-2 cells treated with gp120 (1 μg/mL) for 24 h. Scale bars, 15 μm. **b** Analysis of ZO-1 and E-cadherin fluorescence intensity at cell junctions. **c** Immunoblot analysis of stathmin in Caco-2 cells treated with gp120 for the indicated time. **d** Twenty-four hours after transfection with GFP vector or GFP-stathmin, Caco-2 cells were treated with gp120 for 24 h, and then, the ZO-1 and E-cadherin fluorescence intensity at cell junctions was quantified. **e** Immunoblot analysis of the cells treated with gp120 and MG132 (20 μM) or CQ (25 μM). **f** Immunofluorescence staining of LC3 in the cells treated with gp120 and BFA1 or CQ for 24 h. Scale bar, 20 μm. **g** Immunoprecipitation and immunoblotting results showing the interaction of endogenous p62 with stathmin in RKO cells. **h** Immunoblot analysis of p62, stathmin, and LC3 in the cells transfected with p62 siRNA or scrambled control siRNA and then treated with gp120 for 4 h. **i**, **j** Immunofluorescence staining of ZO-1 and E-cadherin in p62-knockdown cells treated with gp120 for 24 h. Scale bars, 25 μm. **k** Quantification of α-tubulin and acetylated α-tubulin fluorescence in Caco-2 cells treated with gp120 and AMD3100 for 24 h. **l** Immunofluorescence staining of α-tubulin and acetylated α-tubulin in Caco-2 cells 24 h after they were transfected with GFP vector or GFP-tagged stathmin and then treated with gp120. Scale bar, 20 μm. **m** Immunoprecipitation and immunoblotting results showing the interaction of Flag-PLEKHA7 with cell junction proteins in HEK293T cells. Forty hours after transfection with Flag-PLEKHA7 or GFP-CAMSAP3, the cells were treated with gp120 for 6 h. The asterisk indicates nonspecific bands. **n** A model for HIV-1 penetration of the mucosal barrier: (1) gp120 binds to its receptor; (2) stathmin is degraded by autophagy; (3) reduction in stathmin leads to microtubule hyperstabilization; (4) perturbation of microtubule turnover disrupts cell junctions; (5) HIV-1 breaches the mucosal barrier; and (6) HIV-1 reaches and attacks host immune cells. ns, not significant. Data are presented as mean ± SE

We then sought to investigate the mechanism by which gp120 disrupts these cell junctions. Analysis of whole-cell lysates from control and gp120-treated RKO human colon cancer cells revealed that gp120 significantly decreased the level of stathmin (Supplementary Data Fig. S2a), a microtubule-destabilizing protein. The reduction of stathmin by gp120 was confirmed by immunoblotting (Fig. 1c). This deleterious effect on stathmin was abrogated by treatment with AMD3100 (Supplementary Data Fig. S2b), a compound that antagonizes the gp120 interaction with its chemokine coreceptors.^2^ The levels of stathmin were also decreased in HCT116 human colon cancer cells, Ca9-22 human oral cancer cells, and VK2 human vaginal epithelial cells following incubation with gp120 (Supplementary Data Fig. S2c). In contrast, the levels of other microtubule-binding proteins were not significantly altered by gp120 (Supplementary Data Fig. S2d). We also found that the overexpression of stathmin could rescue both tight and adherens junctions that had been disrupted by gp120 treatment (Fig. 1d and Supplementary Data Fig. S2e). These results suggest that the perturbation of epithelial cell junctions by gp120 results from the decrease in the level of stathmin.

To investigate the molecular mechanism underlying the reduction of stathmin, we examined its stability. The half-life of stathmin was decreased in the presence of gp120 (Supplementary Data Fig. S3a, b). Treatment with the autophagy-lysosome inhibitor chloroquine (CQ), but not the proteasome inhibitor MG132, ameliorated the decrease in stathmin, suggesting that stathmin is degraded by the autophagy-lysosome pathway (Fig. 1e). To test this hypothesis, we quantified fluorescent puncta corresponding to the autophagy marker microtubule-associated protein 1A/1B-light chain 3 (LC3) in autophagosomes.^3^ Incubation with gp120 enhanced the formation of intracellular LC3 puncta (Fig. 1f). Accordingly, more stathmin was targeted to intracellular LC3 puncta in the presence of gp120 (Supplementary Data Fig. S3c). Immunoprecipitation analysis revealed the association of green fluorescent protein (GFP)-stathmin with Flag-p62 (Supplementary Data Fig. S3d), the receptor for cargoes destined for degradation by autophagy.^3^ Moreover, treatment with gp120 promoted the interaction between endogenous stathmin and p62 (Fig. 1g). Compared to that of the control cells, the level of stathmin was not changed by gp120 in p62-depleted cells (Fig. 1h). In addition, knocking down Atg5 or Atg6, two other autophagy regulators,^3^ prevented the gp120-induced decrease in stathmin (Supplementary Data Fig. S3e). We also found that the disruption of tight and adherens junctions by gp120 was blocked by silencing Atg5, Atg6, or p62 (Fig. 1i, j and Supplementary Data Fig. S4a–d) or by treating cells with bafilomycin A1 (BFA1), another autophagy inhibitor, or CQ (Supplementary Data Fig. S4e–h). Collectively, these data suggest that the gp120-induced autophagic degradation of stathmin contributes to the breakdown of cell junctions.

We then investigated whether the gp120-induced reduction in stathmin interferes with microtubule turnover. We found that incubating Caco-2 cells with gp120 caused microtubule hyperstabilization, as evidenced by an increase in microtubule acetylation, which was abrogated by treatment with AMD3100 (Fig. 1k and Supplementary Data Fig. S5a). Similar results were obtained with oral and vaginal epithelial cells (Supplementary Data Fig. S5b, c). Microtubules in gp120-treated cells were less affected by cold shock than microtubules in the control cells, which were depolymerized, confirming the microtubule hyperstabilization induced by gp120 (Supplementary Data Fig. S5d, e). We also found that overexpression of stathmin could abrogate the microtubule hyperstabilization induced by gp120 (Fig. 1l). In addition, the gp120-induced increase in microtubule hyperstabilization was blocked by treatment with BFA1 or CQ (Supplementary Data Fig. S6a, b) or upon knockdown of Atg5, Atg6, or p62 (Supplementary Data Fig. S6c–f). Together, these findings suggest that the gp120-induced autophagic degradation of stathmin underlies microtubule hyperstabilization.

To study whether microtubule hyperstabilization contributes to gp120-induced perturbation of cell junctions, we examined the intracellular distribution of the calmodulin-regulated spectrin-associated protein 3 (CAMSAP3)/pleckstrin homology domain-containing A7 (PLEKHA7) complex, which is known to associate with the minus ends of microtubules to recruit various junctional proteins.^4,5^ We found that gp120 decreased the colocalization of CAMSAP3 and PLEKHA7 at the plasma membrane and that this decrease was abolished by treatment with AMD3100 or CQ (Supplementary Data Fig. S7a). In addition, gp120 impaired the junctional colocalization of CAMSAP3 with ZO-1, CAMSAP3 with E-cadherin/p120 catenin, PLEKHA7 with ZO-1/occludin, and PLEKHA7 with E-cadherin/p120 catenin/β-catenin, which was abrogated by treatment with AMD3100 or CQ (Supplementary Data Fig. S7b–d and Supplementary Data Fig. S8a–e). Immunoblotting also indicated that gp120 decreased the levels of membrane-associated CAMSAP3, PLEKHA7, β-catenin, E-cadherin, and ZO-1 (Supplementary Data Fig. S8f). Using immunoprecipitation, we also found that gp120 promoted the interaction of CAMSAP3/PLEKHA7 with β-catenin/p120 catenin, whereas it suppressed the interaction of CAMSAP3/PLEKHA7 with E-cadherin/paracingulin/ZO-1 (Fig. 1m). These results suggest that gp120 disrupts the steady state of junctional protein complexes by altering the stability of CAMSAP3/PLEKHA7-anchored microtubules.

In summary, we demonstrate that autophagic degradation of stathmin is triggered by the interaction of HIV-1 gp120 with mucosal epithelial cells, presumably through gp120 binding to its classic receptors/coreceptors on cell surfaces, although alternative mechanisms may exist. Subsequently, stathmin degradation promotes the hyperstabilization of microtubules and consequently disrupts junctional protein complexes, leading to enhanced mucosal permeability to initiate the infection cycle (Fig. 1n). These findings reveal a previously undescribed mechanism by which HIV-1 exposure breaches epithelial integrity and have important implications in the design of effective strategies to prevent mucosal transmission of HIV-1.

## Supplementary information

Supplementary information
