# Supplementary material for: HIV-1 exposure triggers autophagic degradation of stathmin and hyperstabilization of microtubules to disrupt epithelial cell junctions
Source: Signal Transduct Target Ther. 2020 Jun 19;5:79. doi: 10.1038/s41392-020-0175-1 (PMC7303115; doi:10.1038/s41392-020-0175-1)
Supplement: Supplementary file 1 — Supplementary information [file 41392_2020_175_MOESM1_ESM.pdf]

Supplementary Materials

HIV-1 exposure triggers autophagic degradation of stathmin and hyperstabilization of microtubules to disrupt epithelial cell junctions

Wei Xie[1], Dengwen Li[2,*], Dan Dong[1], Yuanyuan Li[2], You Zhang[2], Liangwei Duan[2], Xinqi Liu[2], Wenxiang Meng[3], Min Liu[1], Jun Zhou[1,2,*]

[1]Institute of Biomedical Sciences, Shandong Provincial Key Laboratory of Animal Resistance Biology, Collaborative Innovation Center of Cell Biology in Universities of Shandong, College of Life Sciences, Shandong Normal University, Jinan, Shandong 250014, China.
[2]State Key Laboratory of Medicinal Chemical Biology, College of Life Sciences, Nankai University, Tianjin 300071, China.
[3]State Key Laboratory of Molecular Developmental Biology, Institute of Genetics and Developmental Biology, Chinese Academy of Sciences, Beijing 100101, China.

*Correspondence: D.L. (email: dwli@nankai.edu.cn) or J.Z. (email: junzhou@sdnu.edu.cn)

This file includes:

Materials and Methods

References for “Materials and Methods” section

Supplementary Figures
Supplementary Figure 1. HIV-1 gp120 induces an acute increase in epithelial permeability.
Supplementary Figure 2. Decrease in the level of stathmin underlies the disruption of cell junctions by gp120.
Supplementary Figure 3. HIV-1 gp120 induces autophagy to stimulate stathmin degradation.
Supplementary Figure 4. HIV-1 gp120-induced autophagy contributes to the disruption of cell junctions.
Supplementary Figure 5. HIV-1 gp120 induces microtubule hyperstabilization.
Supplementary Figure 6. Reversal of gp120-induced microtubule hyperstabilization by autophagy inhibition.
Supplementary Figure 7. HIV-1 gp120 disrupts junctional protein complexes.
Supplementary Figure 8. HIV-1 gp120 perturbs junctional protein complexes.

Materials and Methods

Antibodies, chemicals, and proteins

Antibodies against the following proteins were used for immunostaining or immunoblotting: acetylated α-tubulin, PLEKHA7, and CAMSAP3 (Merck Millipore, Darmstadt, Germany and Sigma-Aldrich, St. Louis, MO, USA); α-tubulin, stathmin, and E-cadherin (Abcam, Cambridge, MA, USA); stathmin, p120 catenin, paracingulin, and occludin (Proteintech, Rosemont, IL, USA); β-actin, GFP, Na/K ATPase, and Flag (Abways, Shanghai, China); p62 and LC3 (MBL, Woburn, MA, USA); β-catenin (ABclonal, Woburn, MA, USA); and ZO-1 (Thermo Fisher Scientific, Waltham, MA, USA). Horseradish peroxidase-conjugated secondary antibodies were from Amersham Biosciences (Piscataway, NJ, USA) and Alexa Fluor 488-, 568- or 647-conjugated secondary antibodies were from Abcam. Protein A/G-agarose beads (Pierce, Rockford, IL, USA), GFP-agarose (MBL), and Flag-agarose (Abmart, Berkeley Heights, NJ, USA) were used for immunoprecipitation. FITC-dextran, AMD3100, MG132, CQ, 4',6-diamidino-2-phenylindole (DAPI), and BFA1 were from Sigma-Aldrich. CHX was from MedChemExpress (Monmouth Junction, NJ, USA). Recombinant HIV-1 gp120 and human CD4 proteins were expressed in stably transfected Drosophila S2 cells and purified by affinity chromatography followed by gel filtration chromatography as described previously.[1]

Cells

Caco-2 cells were cultured in Minimal Essential Medium (Thermo Fisher Scientific). T84 cells were cultured in Dulbecco's Modified Eagle's Medium (DMEM)/F-12 (Thermo Fisher Scientific). SW480 cells were cultured in Roswell Park Memorial Institute 1640 medium (Hyclone, Logan, UT, USA). HCT116, RKO, HT-29, Ca9-22, HOEC, VK2, and HEK293T cells were cultured in DMEM (Hyclone). All cells were cultured in the medium supplemented with 10% fetal bovine serum and 100 U/ml penicillin/streptomycin (both from Biological Industries, Cromwell, CT, USA) in a humidified incubator of 5% CO_2 at 37°C.

Plasmids

Stathmin and p62 cDNAs were amplified by PCR and cloned into the pEGFP-N1 and pCMV5-Flag vectors, respectively. Stathmin deletion and point mutants were generated using the Quick Change Site-Directed Mutagenesis kit (Stratagene, La Jolla, CA, USA). Plasmids for GFP-CAMSAP3 and Flag-PLEKHA7 were described previously.[2] Plasmids were transfected with polyethylenimine (Polysciences, Warrington, PA, USA) for HEK293T cells and with Lipofectamine 3000 (Thermo Fisher Scientific) for other cell lines.

siRNAs

siAtg5 (5'-CAUCUGAGCUACCCGGAUA-3'), siAtg6 (5'-CAGUUUGGCACAAUCAAUA-3'), and sip62 (5'-

GCAUUGAAGUUGAUAUCGAU-3') were synthesized by RiboBio (Guangzhou, China) and transfected using Lipofectamine RNAi-max (Thermo Fisher Scientific).

Immunoblotting

Whole cell lysates were prepared with the Triton X-100 lysis buffer (50 mM Tris-HCl pH7.4, 150 mM NaCl, 1 mM Na_4VO_3, 10 mM NaF, 1 mM EDTA, 1% Triton X-100) supplemented with 1 mM phenylmethylsulfonyl fluoride and a protease inhibitor cocktail (Roche Diagnostics, Indianapolis, IN, USA). Plasma membrane proteins were prepared using a Plasma Membrane Protein Extraction kit (BioVision, Milpitas, CA, USA). Proteins were resolved by SDS-PAGE and transferred onto the Immobilon polyvinylidene difluoride membrane (Merck Millipore). Densitometric analysis of the blots was performed with the ImageJ software (National Institutes of Health, Bethesda, MD, USA).

Immunofluorescence microscopy

Cells grown on glass coverslips were fixed with methanol at -20°C for 5 min (for staining microtubules), or fixed with 4% paraformaldehyde at room temperature for 20 min and then permeabilized with 0.5% Triton X-100 (for staining cell junctions proteins). Cells were then blocked with 4% bovine serum albumin in phosphate-buffered saline (PBS) for 1 h and incubated at 4°C overnight with primary antibodies followed by Alexa Fluor 488-, 568- or 647-conjugated secondary antibodies at room temperature in the dark for 1 h. The cells were stained with DAPI in the dark for 5 min, and coverslips were mounted with 90% glycerol in PBS and examined with a TCS SP8 confocal microscope equipped with the LAS X software (Leica, Wetzlar, Germany). Image acquisition was set to z-series of multiple planes with 0.6-1.0 μm distance and 3-5 μm thickness. The acquired images were converted to 2D images by maximum intensity projection in the z-direction before image analysis. The quantification of fluorescent images was performed using the polyhedral tool of ImageJ. Fluorescence intensity was measured in the selected junctional area or in the whole cell, and the background signal was subtracted. The junctional signal (%) was referred to as the ratio of the fluorescence intensity in the junctional area to the fluorescence intensity in the whole cell. For all fluorescence images, a minimum of 30 cells from repeated measures of independent experiments were quantified.

Measurement of TEER and paracellular permeability

Cells were seeded on transwell inserts with a pore size of 0.4 μm and cultured for 8 days to achieve confluency and then treated with gp120 for 24 h. TEER was recorded using a Millicell ERS-2 voltohmmeter (Merck Millipore), and net TEER was calculated by subtracting the resistance value of the filter alone from the measured values. To evaluate paracellular permeability, cells grown on transwell inserts to confluency were treated with gp120 for 24 h, and 1 mg/mL

FITC-dextran was added to the upper surface of culture inserts. After incubation with FITC-dextran for 4 h, the fluorescence intensity of the lower-layer culture medium was measured using the Synergy 4 microplate reader (BioTek, Winooski, VT, USA), with excitation and emission wavelengths of 485 and 535 nm, respectively.

LC-MS/MS

To identify proteins significantly changed by gp120, RKO cells were treated with 0 or 1 μg/mL gp120 for 6 h, and cell lysates were subjected to LC-MS/MS analysis by PTM Biolabs (Hangzhou, China).

Statistical analysis

Experiments were repeated at least three times. Statistical comparisons were performed using the Prism software (GraphPad, La Jolla, CA, USA). Statistical significance was evaluated with the unpaired two-tailed *t* test or by one-way analysis of variance. Data are presented as mean ± SE. $P < 0.05$ was considered statistically significant.

References

1 Duan, L. W. *et al.* A non-canonical binding interface in the crystal structure of HIV-1 gp120 core in complex with CD4. *Sci. Rep.* **7**, 46733 (2017).

2 Meng, W., Mushika, Y., Ichii, T. & Takeichi, M. Anchorage of microtubule minus ends to adherens junctions regulates epithelial cell-cell contacts. *Cell* **135**, 948-959 (2008).

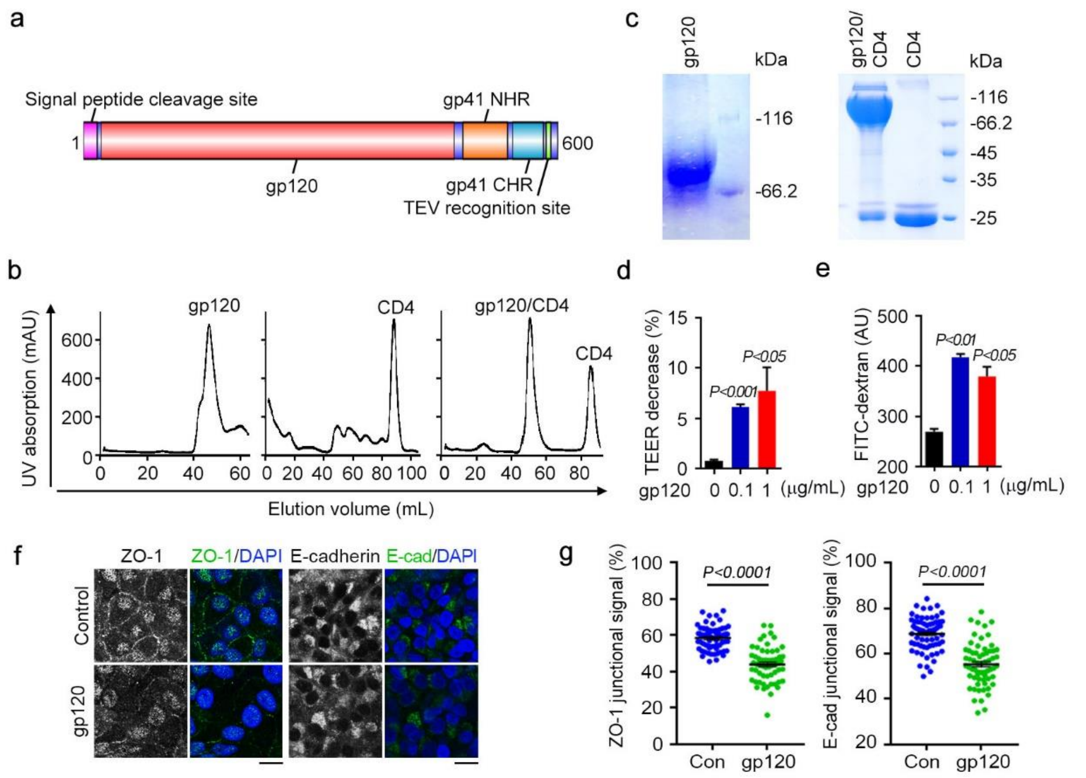

Supplementary Figure 1. HIV-1 gp120 induces an acute increase in epithelial permeability. a Schematic illustration of recombinant gp120. The protein sequence includes a signal peptide cleavage site, amino acids 44–493 of HIV-1 gp120, NHR and CHR domains of HIV-1 gp41, and a tobacco etch virus (TEV) recognition site. **b, c** Gel filtration chromatography of gp120, CD4, and the gp120/CD4 complex (**b**). CD4 represents a fragment of human CD4 containing the N-terminal two immunoglobulin-like domains. The protein solution was collected at the peak point of ultraviolet light absorption at 280 nm and subjected to SDS-PAGE and Coomassie blue staining (**c**). **d** Cells grown to confluency on transwell inserts were treated with gp120, and TEER was measured 24 h later. **e** Cells grown to confluency on transwell inserts were treated with gp120 for 24 h. Paracellular permeability was analyzed by measuring the fluorescence intensity of FITC-dextran that moved to the lower-layer culture medium over a 4-h period. **f** Immunofluorescence staining of ZO-1 and E-cadherin in HOEC cells incubated with 0 or 1 μg/mL gp120 for 24 h. Scale bars, 10 μm. **g** Quantification of ZO-1 and E-cadherin fluorescence intensity at cell junctions based on images from panel (**f**). Data are presented as mean ± SE.

a

Identified proteins	Score	Number of unique peptides	Protein Coverage (%)
stathmin	52	5	34.2
ZNF593	24	2	21.6
C11orf98	20.2	1	17.2
MCRIP1	15.67	1	11.3

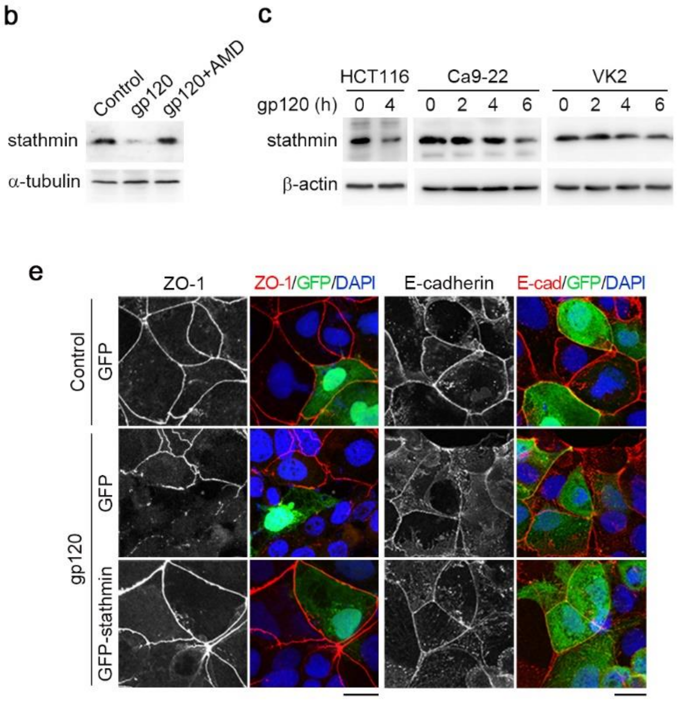

Supplementary Figure 2. Decrease in the level of stathmin underlies the disruption of cell junctions by gp120. a RKO cells were incubated with 0 or 1 μg/mL gp120 for 6 h, and proteins significantly reduced by gp120 were identified by mass spectrometric analysis. **b** Immunoblot analysis of stathmin in RKO cells incubated with 1 μg/mL gp120 and 10 μM AMD3100 for 6 h. **c** Immunoblot analysis of stathmin in cells incubated with 0 or 1 μg/mL gp120 for the indicated time. **d** Immunoblot analysis of microtubule-binding proteins in RKO cells incubated with gp120 for 4 h. **e** Immunofluorescence staining of ZO-1 and E-cadherin in Caco-2 cells transfected with GFP vector or GFP-stathmin for 24 h and then treated with gp120 for 24 h. Scale bars, 25 μm.

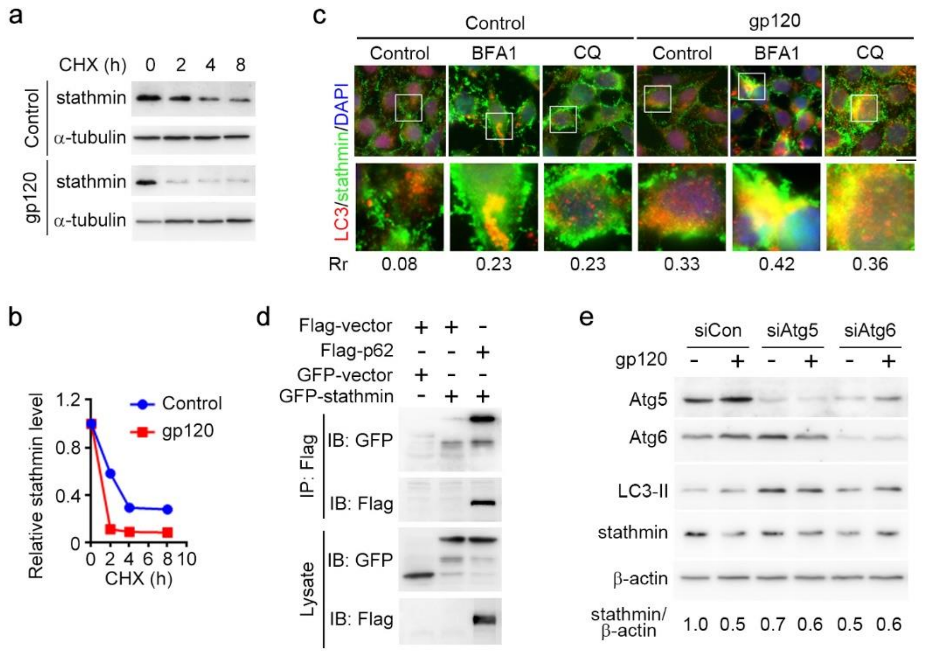

Supplementary Figure 3. HIV-1 gp120 induces autophagy to stimulate stathmin degradation. a Immunoblot analysis of stathmin in cells treated with gp120 for 4 h and then with CHX (100 μg/mL) for the indicated time. **b** Quantification of stathmin levels using the ImageJ software. **c** Subcellular localization of stathmin and LC3 in cells treated with gp120 and BFA1 or CQ. Pearson's correlation coefficient (Rr) was calculated. Scale bar, 10 μm. **d** Immunoprecipitation (IP) and immunoblotting (IB) showing the interaction of Flag-p62 with GFP-stathmin in HEK293T cells. **e** Cells were transfected with control, Atg5, or Atg6 siRNA and then treated with gp120 for 4 h. The level of stathmin relative to β-actin was quantified.

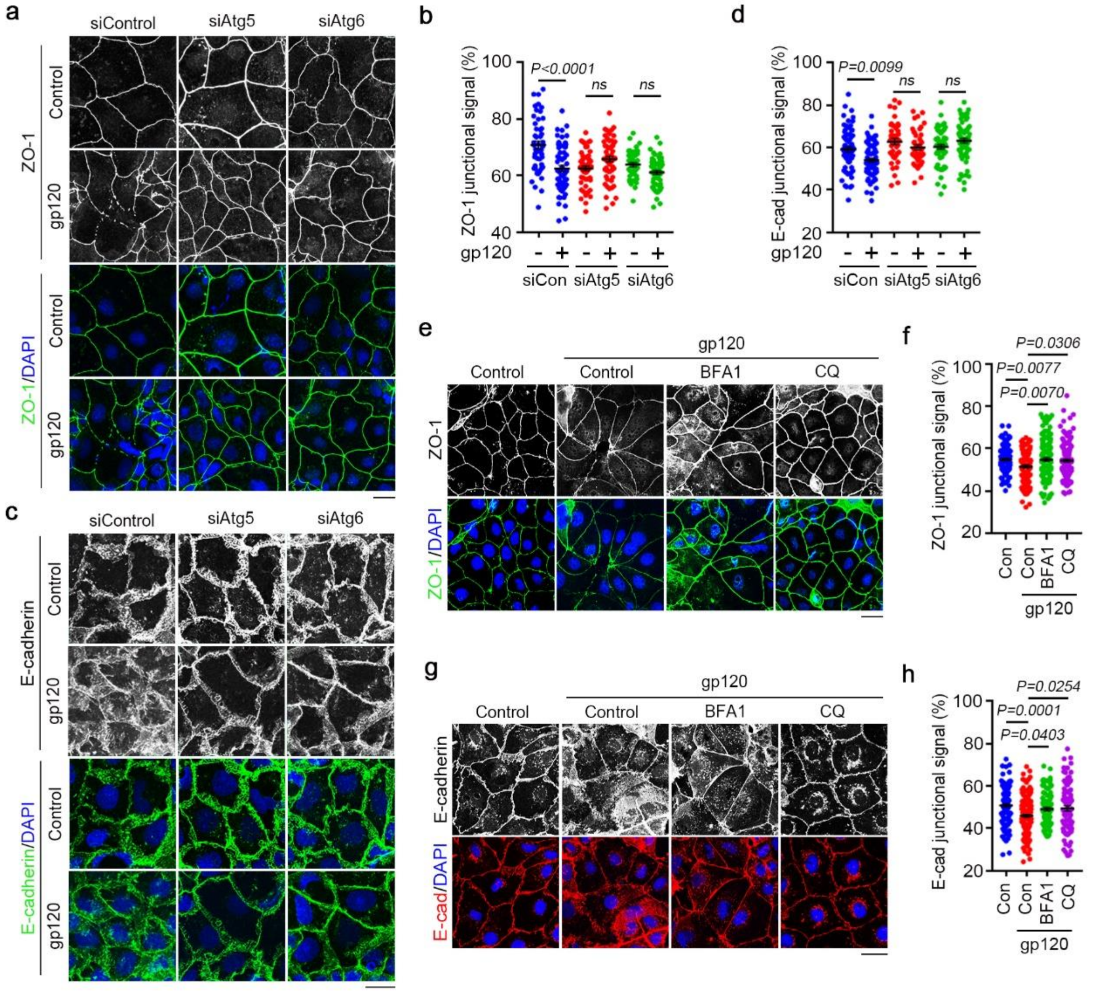

Supplementary Figure 4. HIV-1 gp120-induced autophagy contributes to the disruption of cell junctions. a, c Immunofluorescence staining of ZO-1 (**a**) and E-cadherin (**c**) in cells treated with gp120 for 24 h following Atg5 or Atg6 knockdown. Scale bars, 25 μm. **b, d** Quantification of ZO-1 (**b**) and E-cadherin (**d**) fluorescence intensity at cell junctions based on images from panels (**a**) and (**c**). **e, g** Immunofluorescence staining of ZO-1 (**e**) and E-cadherin (**g**) in cells treated with gp120 and BFA1 or CQ for 24 h. Scale bars, 40 μm. **f, h** Quantification of ZO-1 (**f**) and E-cadherin (**h**) fluorescence intensity at cell junctions based on images from panels (**e**) and (**g**). ns, not significant. Data are presented as mean ± SE.

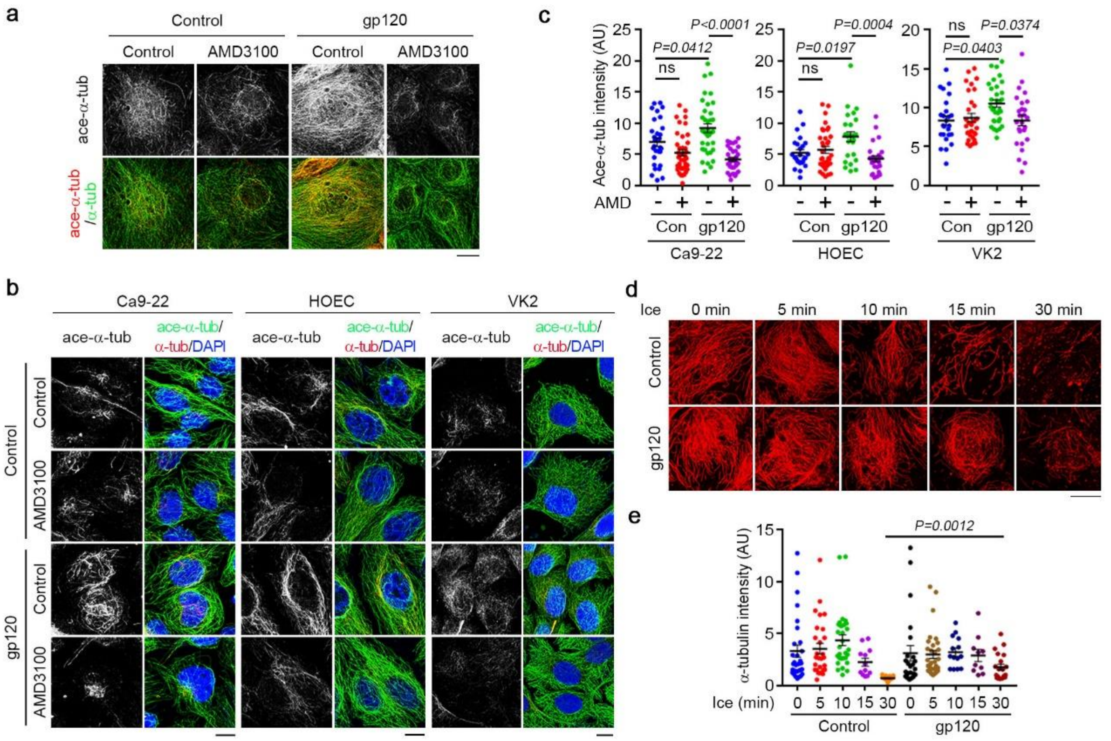

Supplementary Figure 5. HIV-1 gp120 induces microtubule hyperstabilization. a Immunofluorescence staining of α-tubulin and acetylated α-tubulin in Caco-2 cells treated with gp120 and AMD3100 for 24 h. Scale bar, 20 μm. **b** Immunofluorescence staining of α-tubulin and acetylated α-tubulin in cells treated with gp120 and AMD3100 for 24 h. Scale bars, 10 μm. **c** Quantification of the fluorescence intensity of acetylated α-tubulin based on images from panel (**b**). **d** Immunofluorescence staining of α-tubulin in Caco-2 cells placed on ice for the indicated time to induce microtubule depolymerization. Scale bar, 40 μm. **e** Quantification of α-tubulin fluorescence. ns, not significant. Data are presented as mean ± SE.

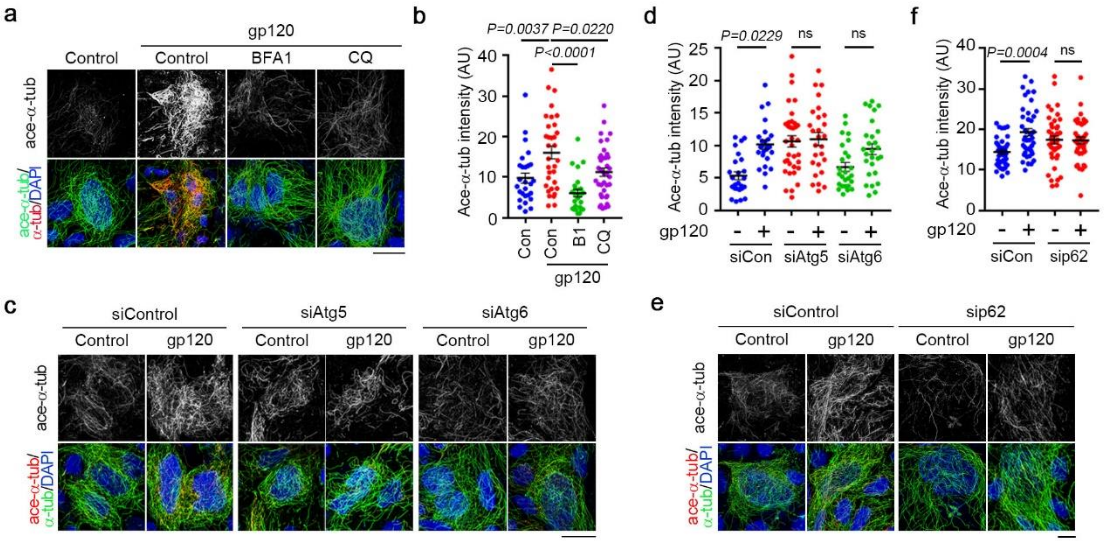

Supplementary Figure 6. Reversal of gp120-induced microtubule hyperstabilization by autophagy inhibition. a Immunofluorescence staining of α-tubulin and acetylated α-tubulin in cells treated with gp120 for 24 h. Scale bar, 20 μm. **b** Quantification of the fluorescence intensity of acetylated α-tubulin based on images from (**a**). **c** Immunofluorescence staining of α-tubulin and acetylated α-tubulin in cells treated with gp120 for 24 h following Atg5 or Atg6 knockdown. Scale bar, 20 μm. **d** Quantification of acetylated α-tubulin fluorescence based on images from panel (**c**). **e** Immunofluorescence staining of α-tubulin and acetylated α-tubulin in cells treated with gp120 for 24 h following p62 knockdown. Scale bar, 10 μm. **f** Quantification of the fluorescence intensity of acetylated α-tubulin based on images from panel (**e**). ns, not significant. Data are presented as mean ± SE.

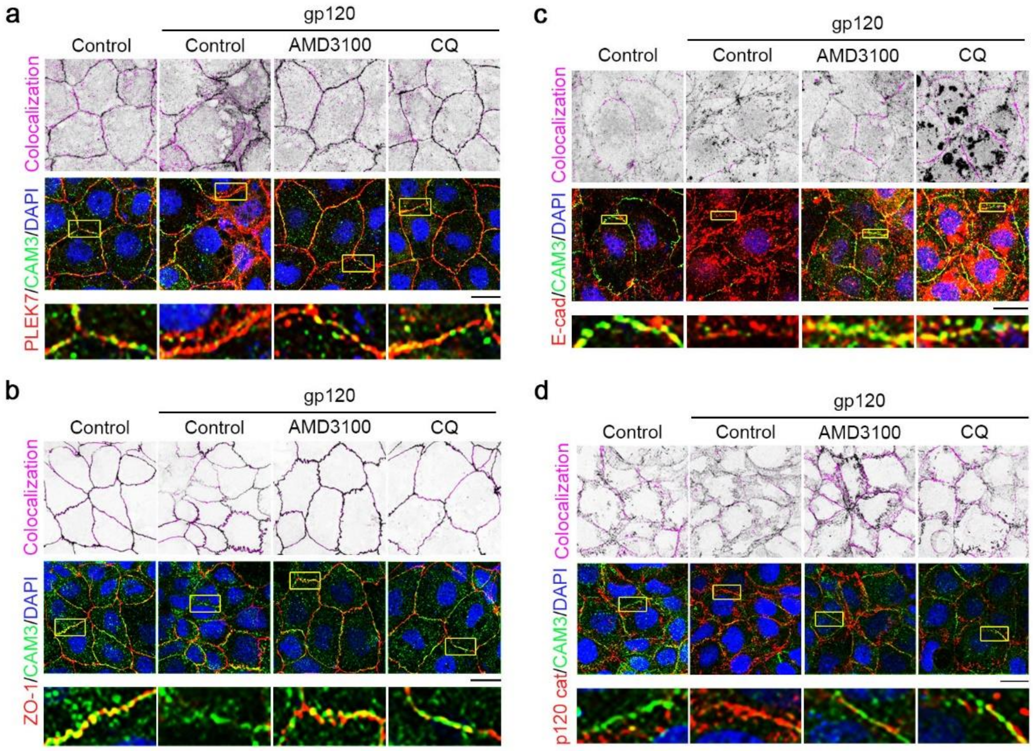

Supplementary Figure 7. HIV-1 gp120 disrupts junctional protein complexes. a Colocalization of PLEKHA7 and CAMSAP3 in Caco-2 cells treated with gp120 and AMD3100 or CQ. Scale bar, 25 μm. Colocalization dots (violet) are shown on the top. **b-d** Colocalization of ZO-1 and CAMSAP3 (**b**), E-cadherin and CAMSAP3 (**c**), and p120 catenin and CAMSAP3 (**d**) in cells treated with gp120 and AMD3100 or CQ. Colocalization dots (violet) are shown on the top. Scale bars, 25 μm.

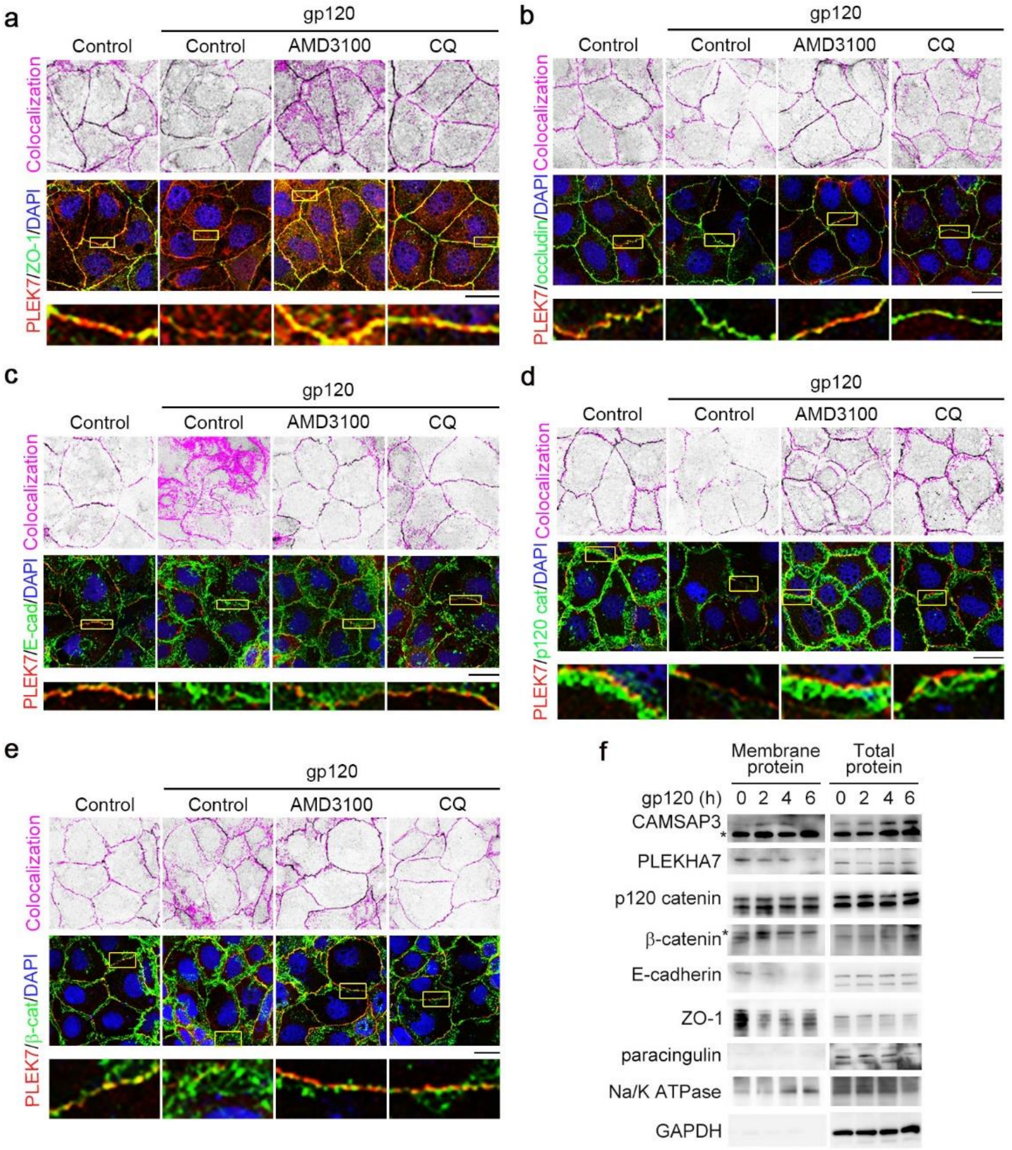

Supplementary Figure 8. HIV-1 gp120 perturbs junctional protein complexes. a-e Colocalization of PLEKHA7 and ZO-1 (**a**), PLEKHA7 and occludin (**b**), PLEKHA7 and E-cadherin (**c**), PLEKHA7 and p120 catenin (**d**), and PLEKHA7 and β-catenin (**e**) in Caco-2 cells treated with gp120 and AMD3100 or CQ. Colocalization dots (violet) are shown on the top. Scale bars, 10 μm. **f** Immunoblot analysis of cell junction proteins in RKO cells treated with gp120 for the indicated time. Na/K ATPase was used as a loading control of membrane proteins. Asterisks indicate nonspecific bands.
